# Serum zinc level independently predicts need for inpatient intubation among patients hospitalized with COVID‐19: A prospective observational study

**DOI:** 10.1002/ncp.70070

**Published:** 2025-11-11

**Authors:** Scott W. McPherson, Frederik J. van Kuijk, Solmaz Ramezani, Susan Vitale, William H. Amundson, Andrew Caraganis, Kathleen S. Mahan, Rusdeep Mundae, Ronald A. Reilkoff, Emily Y. Chew, David A. Wacker

**Affiliations:** ^1^ Department of Ophthalmology and Visual Neurosciences University of Minnesota Medical School Minneapolis Minnesota USA; ^2^ Department of Neurology University of Minnesota Medical School Minneapolis Minnesota USA; ^3^ Department of Neurology University of Nebraska Medical Center Omaha Nebraska USA; ^4^ Division of Epidemiology and Clinical Applications, National Eye Institute National Institutes of Health Bethesda Maryland USA; ^5^ Department of Medicine, Division of Critical Care and Pulmonary Medicine Regions Hospital St. Paul Minnesota USA; ^6^ Department of Medicine, Division of Pulmonary, Allergy, Critical Care, and Sleep Medicine University of Minnesota Medical School Minneapolis Minnesota USA; ^7^ Present address: Department of Ophthalmology & Visual Sciences University of Texas Medical Branch Galveston TX USA

**Keywords:** artificial respiration, COVID‐19, infections, shock, zinc

## Abstract

**Background:**

The coronavirus disease 2019 (COVID‐19) pandemic caused significant morbidity and mortality. Further study of modifiable factors influencing COVID‐19 severity and outcomes continues to be necessary. Serum zinc levels may play a role in modulating COVID‐19 virus replication and consequently influence clinical outcomes.

**Methods:**

This was a prospective observational study of adult patients hospitalized with COVID‐19 assessing the relationship between serum zinc levels and clinical outcomes. Serum zinc levels were measured within 7 days of admission. The primary outcome was the need for intubation at any time during inpatient stay. Secondary outcomes included hospital disposition and incidence of shock and acute kidney injury.

**Results:**

Serum zinc levels could be obtained for 99 patients with COVID‐19. The 25 requiring intubation during hospitalization had a statistically significantly lower median (IQR) zinc concentration (51.6 µg/dl [46.3–62.3 µg/dl]) than those who did not (64.4 µg/dl [55.2–76.0 µg/dl]; *P* < 0.01). Patients requiring more respiratory support on hospital day 1, having acute kidney injury on hospital day 8, or requiring pressors on hospital days 1 or 8 also had significantly lower zinc levels. In multivariable analysis including other clinical factors known to influence outcomes in COVID‐19 disease, serum zinc level remained an independent predictor of the need for intubation (odds ratio 0.941, 95% CI 0.885–0.999; *P* = 0.048).

**Conclusion:**

In multivariable analysis, lower serum zinc level was an independent predictor of inpatient intubation in COVID‐19, but further investigation of zinc supplementation to prevent or reduce severity in COVID‐19 infection is warranted before routine clinical use.

## INTRODUCTION

In the years since it was first detected, coronavirus disease 2019 (COVID‐19) has caused significant morbidity and mortality, contributing to more than one million deaths in the United States alone during its peak from 2020 to 2022.^1^ Although the severity of COVID‐19 has since abated with the development of virus‐specific vaccines and antivirals, vaccine acceptance rates have been suboptimal,^2^ and antivirus therapy effectiveness has been limited by phenomena such as disease rebound.^3^ As such, COVID‐19 still contributed to >48,000 deaths in the US in 2024,^4^ and long‐term ailments following acute COVID‐19 infection (long COVID) continue to affect a significant percentage of those infected.^5^

Zinc deficiency has been shown to be a risk factor for lower respiratory infections, with the World Health Organization (WHO) estimating that it is a major contributor to 16% of lower respiratory infections worldwide.^6^ Furthermore, zinc supplementation has shown promise in preventing lower respiratory infections in developing countries,^7^ although its effectiveness in the treatment of respiratory infections has been variable among those unlikely to be zinc deficient.^8^ Among critically ill patients, zinc levels have generally been shown to be depressed; however, this may not reflect true zinc deficiency but rather result from acute redistribution of zinc from the plasma to the intracellular or interstitial spaces.^9^ Lower zinc levels have been associated with the development of acute respiratory distress syndrome,^10^ although correlation of zinc level with clinical outcomes among those with established respiratory failure has not been demonstrated.^11^

Zinc supplementation has also been suggested to have a beneficial effect for the failure of other organ systems. Patients with chronic liver disease are known to have an increased risk of zinc deficiency owing to alterations in zinc metabolism, including decreased dietary intake and increased urinary excretion,^12^, ^13^ and oral zinc supplementation has been proposed as a beneficial therapy for patients with cirrhosis.^14^, ^15^ Similarly, zinc supplementation has been suggested as an intervention in acute care settings for patients with acute renal failure.^16^

The potential link between zinc, critical illness, and respiratory infections has led to both review and new speculation regarding how zinc could impact COVID‐19 pathogenesis.^17^, ^18^, ^19^ Several observations support the idea that zinc could inhibit key steps in the life cycle of severe acute respiratory syndrome coronavirus 2 (SARS‐CoV‐2). First, the main protease of SARS‐CoV‐2, which is required for viral replication, is inhibited by zinc ions in vitro.^20^ Second, the RNA‐dependent RNA polymerase of the SARS‐CoV virus, which is highly conserved in SARS‐CoV‐2, is inhibited by zinc ions in in vitro and cell culture–based assays.^21^ Because of the structural conservation it is believed that this replication enzyme is also susceptible to inhibition by zinc in SARS‐CoV‐2.^22^ Collectively, these observations led to a proposal that zinc be investigated as a prophylactic or therapeutic agent for COVID‐19 infection.^23^

In clinical studies, lower serum zinc levels have consistently been shown to correlate with COVID‐19 infection, regardless of severity, among both inpatients^24^, ^25^, ^26^, ^27^, ^28^, ^29^, ^30^ and outpatients^31^, ^32^ relative to healthy controls. However, although studies of the relationship between zinc level and disease severity among COVID‐19–infected individuals have generally shown that lower levels predict worsened clinical outcomes,^33^, ^34^, ^35^, ^36^, ^37^, ^38^, ^39^, ^40^, ^41^, ^42^, ^43^, ^44^ this finding has not been universal.^45^, ^46^, ^47^ Of these studies, only a few have used multivariable analysis, and these have shown mixed results with some showing zinc as an independent predictor of severity or mortality^44^, ^48^, ^49^ but others showing no association.^39^, ^50^ Additionally, a genome‐wide association study focusing on genetic mutations known to be associated with lower serum zinc levels did not show them to be associated with COVID‐19 incidence or severity.^51^

In a search for modifiable patient factors affecting disease course and outcome in acute COVID‐19 infection, we performed a prospective observational study of serum zinc levels and clinical outcomes in patients hospitalized with COVID‐19. To assess whether serum zinc level was an independent predictor of COVID‐19 outcomes we used multivariable regression to adjust for other clinical variables that could potentially influence COVID‐19 disease course. We hypothesized that because of the mechanisms described above, higher zinc levels would be protective against disease severity, resulting in an inverse relationship between serum zinc levels and disease severity.

## PATIENTS AND METHODS

### Study design and settings

This was a prospective observational study at four hospitals that examined whether serum zinc level at hospital admission was associated with disease severity and course in COVID‐19 infection. An initial pilot phase was used to provide preliminary estimates (median and IQR; Table S1) of zinc levels in outcome subgroups of interest to allow us to compute minimum sample sizes for detecting differences with a given level of statistical power. We then collected additional data on a larger cohort. Full details are included in Methods S1.

This study was approved by our local institutional review board (STUDY #00009471), which has jurisdiction over all participating hospitals. All procedures followed were in accordance with the ethical standards of the institutional review board. Informed consent was obtained from all patients after the nature of the study's procedures were explained.

### Study population and timeframe

This study recruited patients from July 2020 through June 2022. Full inclusion and exclusion criteria are listed in Methods S1, but the key eligibility criteria were age ≥18 years, hospital admission, and a positive COVID‐19 testing result.

### Data collection

Serum zinc levels were determined within 7 days of admission. All clinical outcome data were extracted from the electronic medical record. The primary outcome for this study was requirement for intubation and mechanical ventilation at any point during hospitalization. Secondary outcomes included respiratory status on hospital day 1 and anytime before hospital day 8, presence of shock on hospital day 1 and anytime before hospital day 8, presence of acute kidney injury on hospital day 1 or anytime before hospital day 8, hospitalization outcome (discharged to home, discharged to facility, or deceased), and occurrence of venous thromboembolism.

To assess patient acuity, data were collected to calculate Sequential Organ Failure Assessment (SOFA) scores; however, serum bilirubin was not measured for most patients, so a modified SOFA (mSOFA) score including the cardiac, neurologic, hematologic, renal, and respiratory subscores, but excluding the hepatic subscore, was used. When partial pressures of arterial oxygen were unavailable they were estimated from peripheral oxygen saturation using the EPIC II study^52^ estimation table (Table S2).

### Statistical analysis

Using preliminary data on serum zinc levels from our pilot group, and assuming that approximately one patient in three would require intubation (also observed during pilot data collection), one‐way analysis of variance analysis (POWER procedure, SAS 9.4) indicated that a total of 75 patients would be required to show a statistically significant difference in serum zinc levels between the intubated and unintubated groups with ≥80% power. We therefore set our enrollment target at 105 patients to allow for attrition.

Comparison of zinc levels between outcomes was performed using Wilcoxon rank sum tests. Tests of correlation were performed by calculating Spearman rank correlation coefficients and associated *P* values. For multivariable analyses, our outcomes were dichotomous so we used logistic regression models. SAS 9.4 was used for all analyses.

## RESULTS

**Figure 1:**
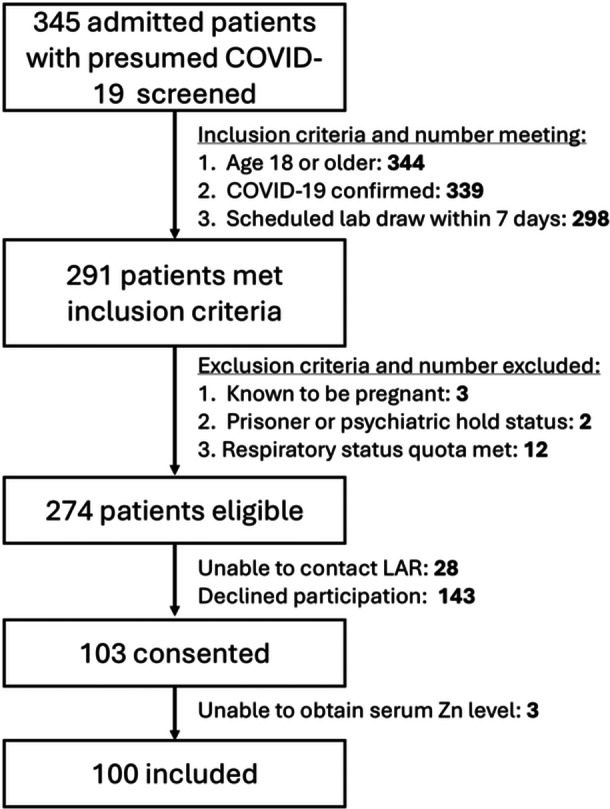
Patient enrollment flow diagram for COVID‐19–infected patients. Enrollments were initially limited by quotas based on respiratory status resulting in exclusion of 12 patients; see Methods S1 for full details. LAR, legally authorized representative; Zn, zinc.

Of 345 screened patients with positive COVID‐19 testing results, we enrolled 103 patients, of whom we were able to obtain serum zinc levels and outcome data on 99 (Figure 1). Patient baseline characteristics and characteristics of hospital course are shown in Table 1.

**Table 1:** Patient demographic information.

Demographic feature	Study cohort values
*N*	100
Age, median (IQR), years	59.5 (45.0–70.0)
Male sex assigned at birth, *n* (%)	61 (61)
Race, *n* (%)	
African or African‐American	10 (10)
Asian	7 (7)
White	74 (74)
Declined to state, unknown, or other	9 (9)
Ethnicity, *n* (%)	
Latino/Latina	9 (9)
Non‐Latino/Latina	90 (90)
Not stated	1 (1)
Medical history, *n* (%)	
Hypertension	53 (53)
Diabetes (type 1 or 2)	30 (30)
Pre‐existing lung pathology	22 (22)
Inhaled tobacco use (any amount), *n* (%)	
Never	68 (68)
Former user	27 (27)
Current user	5 (5)
Heavy alcohol use (more than three drinks per day), *n* (%)	
Never	94 (94)
Previously	3 (3)
Currently	3 (3)
Hydroxychloroquine as a home medication, *n* (%)	2 (2)
Duration of COVID‐19 symptoms^a^ before hospital admission, median (IQR), days	7 (5–10)
Hospital length of stay, mean ± SD, days	12.8 ± 15.0^b^
ICU length of stay, mean ± SD, days	7.1 ± 15.2^b^
Length of mechanical ventilation, mean ± SD, days	6.3 ± 14.9^b^

^a^ Defined as rhinorrhea/nasal congestion, sinus pain, cough, fevers, diarrhea, loss of taste/smell, and/or fatigue.

^b^ Unable to obtain these data for one patient; *n* = 99.

### Univariable analysis of serum zinc level in COVID‐19

Patients meeting the primary outcome of requiring intubation during their hospitalization (*n* = 25) had significantly lower median (IQR) serum zinc levels than those who did not require intubation (*n* = 74) (requiring 51.6 µg/dl [46.3–62.3 µg/dl] µg/dl vs not requiring 64.4 µg/dl [55.2–76.0 µg/dl]; *P* < 0.01; Table 2). Similarly, there was a significant difference in median (IQR) serum zinc levels by hospital day 1 respiratory status (intubated 51.2 µg/dl [46.2–62.9 µg/dl], noninvasive positive pressure ventilation or supplemental oxygen 64.4 µg/dl [54.2–74.3 µg/dl], room air 59.5 µg/dl [52.2–80.6 µg/dl]; *P* = 0.02). Although serum zinc levels were lower for those on room air than those needing noninvasive positive pressure ventilation or supplemental oxygen, a dichotomous comparison between these groups did not show statistical significance (*P* = 0.80).

**Table 2:** Serum zinc level by primary and secondary outcomes in COVID‐19–infected patients.

Outcome	*n*	Serum zinc level, median (IQR), µg/dl	*P* value
Full cohort	100	61.8 (51.8–73.3)	–
Primary outcome			
Need for mechanical ventilation at any point during hospitalization (*n* = 99^a^)			
Yes	25	51.6 (46.3–62.3)	<0.01
No	74	64.4 (55.2–76.0)	
Secondary outcomes			
Hospitalization outcome (*n* = 99^a^)			
Discharged home	71	64.0 (53.5–76.0)	0.06
Discharged to medical facility	19	55.2 (44.7–71.7)	
Deceased	9	54.1 (50.2–59.1)	
Respiratory status on hospital day 1			
Intubated	20	51.2 (46.2–62.9)	0.02
Supplemental O_2_ or NIPPV	67	64.4 (54.2–74.3)	
Room air	13	59.5 (52.2–80.6)	
Pressor need by hospital day 1			
Yes	12	50.4 (44.3–64.2)	0.04
No	88	63.4 (53.2–74.5)	
AKI by hospital day 1			
Yes	18	62.7 (53.4–74.8)	0.14
No	82	56.7 (49.2–70.8)	
Respiratory status on hospital day 8			
Intubated	18	54.9 (50.2–66.0)	0.06
Supplemental O_2_ or NIPPV	35	59.6 (51.4–67.2)	
Room air	47	67.4 (55.2–80.2)	
Pressor need by hospital day 8			
Yes	21	51.6 (46.3–62.3)	<0.01
No	79	64.3 (54.2–74.8)	
AKI by hospital day 8			
Yes	26	51.8 (44.7–66.0)	<0.01
No	74	64.2 (55.0–77.1)	
Occurrence of VTE during hospitalization (*n* = 99^a^)			
Yes	8	51.3 (70.7–80.8)	0.47
No	91	51.6 (61.2–72.0)	

Abbreviations: AKI, acute kidney injury; NIPPV, noninvasive positive pressure ventilation; O_2_, oxygen; VTE, venous thromboembolism.

^a^ For these outcomes, data from a single patient were lost because of a clerical error.

There were also significant median (IQR) differences between those requiring pressors and those not on both hospital days 1 (requiring 50.4 µg/dl [44.3–64.2 µg/dl] vs not 63.4 µg/dl [53.2–74.5 µg/dl]; *P* = 0.04) and 8 (requiring 51.6 µg/dl [46.3–62.3 µg/dl] vs not 64.3 µg/dl [54.2–74.8 µg/dl]; *P* < 0.01) and those with and without acute kidney injury on hospital day 8 (with 51.8 µg/dl [44.7–66.0 µg/dl] vs without 64.2 µg/dl [55.0–77.1 µg/dl]; *P* < 0.01). Serum zinc level correlated inversely with length of hospital stay, length of intensive care unit (ICU) stay and duration of mechanical ventilation (Table 3). Interestingly, among the patients for whom serum C‐reactive protein and ferritin levels were measured, these known markers of inflammation correlated poorly with serum zinc levels (Table S3).

**Table 3:** Correlation between serum zinc level, hospital length of stay, ICU length of stay, and mechanical ventilation time in COVID‐19–infected patients.

	Serum zinc level	Hospital length of stay	ICU length of stay	Duration of mechanical ventilation
Serum zinc level				
Spearman correlation coefficient	1.00000	−0.39024	−0.26993	−0.30808
Prob > |r| under H0: ρ = 0	–	<0.0001	0.0069	0.0019
*n*	100	99	99	99
Hospital length of stay				
Spearman correlation coefficient	−0.39024	1.00000	0.72833	0.69044
Prob > |r| under H0: ρ = 0	<0.0001	–	<0.0001	<0.0001
*n*	99	99	99	99
ICU length of stay				
Spearman correlation coefficient	−0.26993	0.72833	1.00000	0.91777
Prob > |r| under H0: ρ = 0	0.0069	<0.0001	–	<0.0001
*n*	99	99	99	99
Duration of mechanical ventilation				
Spearman correlation coefficient	−0.30808	0.69044	0.91777	1.00000
Prob > |r| under H0: ρ = 0	0.0019	<0.0001	<0.0001	–
*n*	99	99	99	99

Abbreviation: ICU, intensive care unit.

### Multivariable analysis of serum zinc level in COVID‐19

In multivariable analysis, serum zinc level was a statistically significant predictor of need for inpatient intubation with higher zinc levels indicating lower odds of intubation (odds ratio [OR] = 0.941, 95% CI 0.885–0.999, *P* = 0.048; for every 1‐µg/dl increase in serum zinc level, the odds of needing intubation decreased by 5.9%; Table 4); however, it was not an independent predictor of hospitalization outcomes of death (OR = 1.006, 95% CI 0.9357–1.082, *P* = 0.874; Table 5) or discharge to home (OR = 0.991, 95% CI 0.949–1.034, *P* = 0.675; Table S4). As a measure of disease acuity at enrollment, an mSOFA score as defined in the Methods section was included in the multivariable models. The mSOFA score was a significant predictor of all three outcomes, with higher mSOFA scores indicating increased odds of intubation and in‐hospital death but decreased odds of discharge home (need for intubation OR = 2.940, 95% CI 1.694–5.101, *P* < 0.001; death OR = 1.587, 95% CI 1.128–2.232, *P* = 0.008; discharge home OR = 0.586, 95% CI 0.447–0.767, *P* < 0.001). Additionally, age was a significant predictor of the need for intubation (OR = 0.936, 95% CI 0.879–0.999, *P* = 0.048; higher age lowers the odds of intubation) and discharge to home (OR = 0.956, 95% CI 0.917–0.998, *P* = 0.038; higher age lowers the odds of discharge to home). Aside from the mSOFA score, history of diabetes was the only statistically significant predictor of hospitalization outcome of death (OR = 18.069, 95% CI 1.500–217.695, *P* = 0.023; diabetes increases the odds of in‐hospital death).

**Table 4:** Multivariable analysis of factors contributing to the need for intubation during hospital stay in COVID‐19–infected individuals.

Covariate	OR (95% CI)	*P* value
Serum zinc level	0.941 (0.885–0.999)	0.048
Age	0.936 (0.879–0.998)	0.042
Male sex	0.823 (0.126–5.402)	0.840
Non‐White race	0.303 (0.038–2.435)	0.262
mSOFA score	2.940 (1.694–5.101)	<0.001
History of hypertension	0.486 (0.063–3.750)	0.489
History of diabetes mellitus (type 1 or 2)	5.005 (0.569–44.004)	0.147
History of lung disease	0.052 (0.001–2.013)	0.113

Abbreviations: mSOFA, modified Sequential Organ Failure Assessment; OR, odds ratio.

**Table 5:** Multivariable analysis of factors contributing to death as the outcome of hospital stay in COVID‐19–infected individuals.

Covariate	OR (95% CI)	*P* value
Serum zinc level	1.006 (0.935–1.082)	0.874
Age	1.069 (0.991–1.153)	0.086
Male sex	0.789 (0.110–5.656)	0.813
Non‐White race	0.413 (0.037–4.645)	0.474
mSOFA score	1.587 (1.128–2.232)	0.008
History of hypertension	0.456 (0.028–7.435)	0.581
History of diabetes mellitus (type 1 or 2)	18.069 (1.500–217.695)	0.023
History of lung disease	0.313 (0.023–4.283)	0.384

Abbreviations: mSOFA, modified Sequential Organ Failure Assessment; OR, odds ratio.

## DISCUSSION

Because of the observed connections between zinc deficiency and lower respiratory infections, as well as the observed in vitro effects of zinc on SARS‐CoV‐2 replication machinery, serum zinc level has been proposed as a modifiable factor influencing disease severity in COVID‐19.^23^ Prior clinical studies have largely supported this hypothesis, with single‐variable analyses showing a correlation between lower serum zinc levels and worsened COVID‐19 disease.^33^, ^34^, ^35^, ^36^, ^37^, ^38^, ^39^, ^40^, ^41^, ^42^, ^43^, ^44^ Our findings in univariable analysis agree well with these prior studies, demonstrating that lower serum zinc levels at admission were associated with increased incidence of endotracheal intubation,^37^, ^44^ shock,^44^ and acute renal failure as well as increased duration of hospitalization,^35^, ^49^ ICU stay, and mechanical ventilation.

Although this may indicate that the serum zinc level is an independent variable in determining COVID‐19 severity, it may also be that the relationship is due in part to other factors. Older age, for example, is associated with increased COVID‐19 mortality^53^, ^54^ and similarly correlates with lower serum zinc levels.^44^ We therefore employed multivariable analysis examining serum zinc level with other clinical factors known to influence COVID‐19 severity. In these analyses, serum zinc level remained a statistically significant predictor of need for intubation during hospitalization. One other study examined serum zinc level as an independent predictor of inpatient intubation need and found it was not; however, it was found to be a predictor of ICU admission.^44^ Our study may have had heightened sensitivity to detect predictors of inpatient intubation because it was specifically powered to assess this outcome. Although one other study found serum zinc level to predict inpatient mortality,^49^ we did not find this to be the case, nor did two other studies employing multivariable analysis.^48^, ^50^

Observational studies and clinical trials of zinc therapy have not shown a conclusive benefit. Among hospitalized patients in one retrospective observational study, patients receiving zinc supplementation along with hydroxychloroquine and azithromycin had a significant reduction in ICU admissions and in‐hospital mortality relative to those receiving only hydroxychloroquine and azithromycin.^55^ However, in a 1:1:1:1 randomized controlled trial comparing zinc, vitamin D, or zinc and vitamin D with placebo among hospitalized patients, zinc did not lead to significant changes in hospital length of stay, mortality, or need for positive pressure ventilation.^56^ Among outpatients, studies of zinc supplementation to prevent COVID‐19 infection in healthy patients have shown conflicting results,^57^, ^58^ and the use of zinc supplementation to mitigate disease course among those infected with COVID‐19 was not shown in a clinical trial to lead to faster symptom resolution or reductions in rates of hospital admission or death.^59^

The discrepancy between observational results, which largely show a correlation between zinc levels and COVID‐19 outcomes, and therapeutic trials, which show limited benefit of zinc supplementation, may be for several reasons. It may be that the timing and delivery route of zinc therapy influence effectiveness. Administration of zinc by mouth did not result in a statistically significant increase in serum zinc levels relative to the control group among hospitalized patients.^56^ Additionally, prior studies of oral zinc supplementation for other indications have shown that only small to moderate increases in serum zinc level occur after prolonged supplementation.^60^, ^61^ It is possible that delivery of zinc by an intravenous route could more aggressively raise zinc levels^62^ yielding different clinical results, but clinical trials to demonstrate the feasibility and efficacy of such a therapy regimen would be needed before routine clinical use.

It may also be that the observed correlations between serum zinc level and clinical outcomes are noncausative, perhaps resulting from a normal physiologic stress response, limiting the role for zinc supplementation as a possible prophylaxis or therapy. The interaction between zinc metabolism and systemic inflammation makes assessment of this possibility challenging, especially because most studies are constrained to measurements of serum zinc level obtained only after the onset of acute illness. Although true zinc deficiency has been associated with deleterious immunologic effects,^63^, ^64^ exposure to acute inflammatory processes such as acute respiratory distress syndrome^10^ or sepsis^65^ causes a reactive decrease in serum zinc levels regardless of baseline deficiency as zinc is redistributed intracellularly.^64^ Interestingly, rather than a clear inverse correlation, serum zinc levels in our study correlated poorly with classical markers of acute inflammation. Nonetheless, this does not establish a causal relationship between serum zinc levels and clinical outcomes and it may be that serum zinc levels are simply a marker for disease severity with more serious disease‐lowering zinc levels because of poor intake, increased losses, high metabolic demand, or the effects of noninflammatory systemic stress.

Interestingly, in multivariable analysis, increasing patient age showed a statistically significant reduction in the OR of requiring inpatient intubation. This is in contrast with other studies showing that advancing age is associated with COVID‐19 severity^53^, ^54^ and with our own results showing that the OR of death trends higher with older age, although the effect does not reach statistical significance. One explanation for this finding may be that older patients were more likely to have restrictions on life‐sustaining interventions, including intubation. Although we did not directly collect data on the incidence of Do Not Intubate orders in our study population, associations between age and restrictions on life‐sustaining cares including intubation have been widely observed.^66^, ^67^, ^68^

The timing of serum zinc level testing was a major limitation in our study. Although we would ideally have surveyed serum zinc levels at a uniform point in each patient's disease course, our ability to do so was limited by two factors. First, the interval from a patient's first experience of symptoms to the time that they were admitted to the hospital was variable (7 [5–10] days, median [IQR]). Second, there was some variation between patients of the hospital day on which we sampled serum zinc level. Although our protocol allowed sampling at any point within 7 days of admission, 70% of our patients were sampled by hospital day 3, and 94% by day 4. Although we believe serum zinc levels to be relatively static during this period regardless of any intervention,^62^ we cannot formally rule out transient variations associated with any particular treatment or the infection itself. Additionally, prior studies have demonstrated that serum zinc levels can vary somewhat throughout the day.^69^, ^70^ Although we did not have full control over the time of day that samples were drawn for this study, the vast majority of patients (89%) had zinc levels sampled during morning laboratory rounds.

Our study had several other limitations. First, its observational nature limits our ability to robustly draw conclusions about causal relationships. Second, although our study had multiple sites, they were all in the same geographic area, which limits the diversity of the patient population. This can impact the generalizability of our results to outside areas in ways that are difficult to predict.^71^, ^72^, ^73^ Third, our data were collected over a period of 2 years during which there were significant changes in the clinical approach to COVID‐19 treatment, including the development of effective vaccines; this may have influenced outcomes. Additionally, the relationship between zinc and COVID‐19 severity may not be preserved in future strains.^62^ Finally, given the large number of factors influencing zinc metabolism, we were unable to collect data on every potential variable affecting serum zinc levels in study participants. Although some variables, such as fasting state and obesity, have been shown in healthy patients to have minimal impact on serum zinc levels,^69^ their effect on zinc levels in hospitalized patients may be less predictable. Additionally, the generalizability of our results to populations with conditions that may affect zinc metabolism, such as chronic liver disease, is limited.

Our study demonstrates an inverse correlation between serum zinc level and severity of COVID‐19 disease; however, the wide array of factors influencing zinc metabolism, the absence of positive clinical trials to date, and the inability to derive causality from an observational study all speak to a conservative approach in interpreting these results, and further clinical trials are needed to determine their clinical relevance. Although COVID‐19 has taken a milder form in recent years, should a more virulent strain again arise it would be reasonable to continue clinical trials of zinc supplementation to prevent severe COVID‐19 disease, potentially targeting a sicker inpatient population and using parenteral routes of supplementation. In the absence of such trials though, there is presently insufficient evidence to support the use of zinc supplementation to treat or prevent COVID‐19 infection in routine clinical practice.

## CONCLUSIONS

In this observational study of inpatients with COVID‐19, lower serum zinc level was an independent predictor of need for inpatient intubation in a multivariable model incorporating other known predictors of severity in COVID‐19. Although this may imply a therapeutic role for zinc supplementation in preventing severe COVID‐19 disease, causality cannot be determined from an observational study, and further clinical trials are needed to determine if and how zinc supplementation can be an effective prophylaxis or treatment for COVID‐19.

## FUNDING INFORMATION

This study funding was provided by the Minnesota Lions Vision Foundation. This work was also supported by the University of Minnesota Clinical and Translational Science Institute (grant number UL1TR002494 from the National Institutes of Health's National Center for Advancing Translational Sciences) in the form of access to REDCap database software. Emily Y. Chew and Susan Vitale are supported by funds from the Intramural Research Program at the National Eye Institute, National Institutes of Health.

## AUTHOR CONTRIBUTIONS

Scott W. McPherson contributed to study conception, data interpretation, and manuscript preparation. Frederik J. van Kuijk contributed to study conception, patient enrollment, data collection, data interpretation, and manuscript preparation. Solmaz Ramezani contributed to data collection, data interpretation, and manuscript preparation. Susan Vitale contributed to statistical analysis, data interpretation, and manuscript preparation. William H. Amundson contributed to patient enrollment, data collection, and manuscript preparation. Andrew Caraganis contributed to patient enrollment, data collection, and manuscript preparation. Kathleen S. Mahan contributed to patient enrollment, data collection, and manuscript preparation. Rusdeep Mundae contributed to patient enrollment, data collection, and manuscript preparation. Ronald A. Reilkoff contributed to patient enrollment, data collection, and manuscript preparation. Emily Y. Chew contributed to data interpretation and manuscript preparation. David A. Wacker contributed to study conception, patient enrollment, data collection, data interpretation, and manuscript preparation. As Principal Investigator, David A. Wacker had full access to all data in the study and takes responsibility for the integrity of the data and accuracy of the data analysis.

## CONFLICT OF INTEREST STATEMENT

None declared.

## Supporting information


**Table S1:** Pilot data primary outcome statistics. **Table S2:** Estimation of PaO_2_ from measured SpO_2_ (EPIC II study estimation table). **Table S3:** Correlation between serum zinc level, and levels of C‐reactive protein and ferritin. **Table S4:** Multivariable analysis of factors contributing to discharge to home as the outcome of hospital stay in COVID‐19 infected individuals.

McPherson 2025.

## References

[1] Ahmad FB , Cisewski JA , Xu J , Anderson RN . COVID‐19 mortality update—United States, 2022. MMWR Morb Mortal Wkly Rep. 2023;72(18):493‐496. 37141157 10.15585/mmwr.mm7218a4 PMC10168601

[2] Lazarus JV , Wyka K , White TM , et al. A survey of COVID‐19 vaccine acceptance across 23 countries in 2022. Nature Med. 2023;29(2):366‐375. 36624316 10.1038/s41591-022-02185-4

[3] Edelstein GE , Boucau J , Uddin R , et al. SARS‐CoV‐2 virologic rebound with nirmatrelvir‐ritonavir therapy: an observational study. Ann Intern Med. 2023;176(12):1577‐1585. 37956428 10.7326/M23-1756 PMC10644265

[4] US Centers for Disease Control and Prevention COVID data tracker. Accessed February 11 2025. https://covid.cdc.gov/covid-data-tracker

[5] Mandel H , Yoo YJ , Allen AJ , et al. Long‐COVID incidence proportion in adults and children between 2020 and 2024: an electronic health record‐based study from the RECOVER initiative. Clin Infect Dis. 2025;80(6):1247‐1261. 39907495 10.1093/cid/ciaf046 PMC12272849

[6] World Health Organization . The World Health Report 2002: Reducing Risks, Promoting Healthy Life. World Health Organization; 2002.

[7] Roth DE , Richard SA , Black RE . Zinc supplementation for the prevention of acute lower respiratory infection in children in developing countries: meta‐analysis and meta‐regression of randomized trials. Int J Epidemiol. 2010;39(3):795‐808. 20156999 10.1093/ije/dyp391

[8] Hunter J , Arentz S , Goldenberg J , et al. Zinc for the prevention or treatment of acute viral respiratory tract infections in adults: a rapid systematic review and meta‐analysis of randomised controlled trials. BMJ Open. 2021;11(11):e047474. 10.1136/bmjopen-2020-047474 PMC8578211 34728441

[9] Koekkoek WAC , van Zanten ARH . Antioxidant vitamins and trace elements in critical illness. Nutr Clin Pract. 2016;31(4):457‐474. 27312081 10.1177/0884533616653832

[10] Boudreault F , Pinilla‐Vera M , Englert JA , et al. Zinc deficiency primes the lung for ventilator‐induced injury. JCI Insight. 2017;2(11):e86507. 28570269 10.1172/jci.insight.86507 PMC5453708

[11] Linko R , Karlsson S , Pettilä V , et al. Serum zinc in critically ill adult patients with acute respiratory failure. Acta Anaesthesiol Scand. 2011;55(5):615‐621. 21827444 10.1111/j.1399-6576.2011.02425.x

[12] Grüngreiff K , Reinhold D , Wedemeyer H . The role of zinc in liver cirrhosis. Ann Hepatol. 2016;15(1):7‐16. 26626635 10.5604/16652681.1184191

[13] Ullah MI , Alameen AAM , Al‐Oanzi ZH , et al. Biological role of zinc in liver cirrhosis: an updated review. Biomedicines. 2023;11(4):1094. 37189711 10.3390/biomedicines11041094 PMC10135863

[14] Mohammad MK , Zhou Z , Cave M , Barve A , McClain CJ . Zinc and liver disease. Nutr Clin Pract. 2012;27(1):8‐20. 22307488 10.1177/0884533611433534 PMC6027651

[15] Grüngreiff K , Reinhold D , Maret W . Why a pinch of zinc in liver disease matters. Ann Hepatol. 2024;29(1):101152. 37704065 10.1016/j.aohep.2023.101152

[16] Xia W , Li C , Zhao D , et al. The impact of zinc supplementation on critically ill patients with acute kidney injury: a propensity score matching analysis. Front Nutr. 2022;9:894572. 35769374 10.3389/fnut.2022.894572 PMC9234667

[17] Skalny A , Rink L , Ajsuvakova O , et al. Zinc and respiratory tract infections: perspectives for COVID‐19 (review). Int J Mol Med. 2020;46(1):17‐26. 32319538 10.3892/ijmm.2020.4575 PMC7255455

[18] Mayor‐Ibarguren A , Busca‐Arenzana C , Robles‐Marhuenda Á . A hypothesis for the possible role of zinc in the immunological pathways related to COVID‐19 infection. Front Immunol. 2020;11:1736. 32754165 10.3389/fimmu.2020.01736 PMC7365859

[19] Wessels I , Rolles B , Rink L . The potential impact of zinc supplementation on COVID‐19 pathogenesis. Front Immunol. 2020;11:1712. 32754164 10.3389/fimmu.2020.01712 PMC7365891

[20] Panchariya L , Khan WA , Kuila S , et al. Zinc(2+) ion inhibits SARS‐CoV‐2 main protease and viral replication in vitro. Chem Commun. 2021;57(78):10083‐10086. 10.1039/d1cc03563k 34514483

[21] te Velthuis AJW , van den Worm SHE , Sims AC , Baric RS , Snijder EJ , van Hemert MJ . Zn(2+) inhibits coronavirus and arterivirus RNA polymerase activity in vitro and zinc ionophores block the replication of these viruses in cell culture. PLoS Pathog. 2010;6(11):e1001176. 21079686 10.1371/journal.ppat.1001176 PMC2973827

[22] Pormohammad A , Monych N , Turner R . Zinc and SARS‐CoV‐2: a molecular modeling study of Zn interactions with RNA‐dependent RNA‐polymerase and 3C‐like proteinase enzymes. Int J Mol Med. 2020;47(1):326‐334. 33236142 10.3892/ijmm.2020.4790 PMC7723401

[23] McPherson SW , Keunen JE , Bird AC , Chew EY , van Kuijk FJ . Investigate oral zinc as a prophylactic treatment for those at risk for COVID‐19. Am J Ophthalmol. 2020;216:A5‐A6. 32505362 10.1016/j.ajo.2020.04.028 PMC7247979

[24] Anuk AT , Polat N , Akdas S , et al. The relation between trace element status (zinc, copper, magnesium) and clinical outcomes in COVID‐19 infection during pregnancy. Biol Trace Elem Res. 2021;199(10):3608‐3617. 33236293 10.1007/s12011-020-02496-y PMC7685187

[25] Elham AS , Azam K , Azam J , Mostafa L , Nasrin B , Marzieh N . Serum vitamin D, calcium, and zinc levels in patients with COVID‐19. Clin Nutr ESPEN. 2021;43:276‐282. 34024527 10.1016/j.clnesp.2021.03.040 PMC8053215

[26] Jothimani D , Kailasam E , Danielraj S , et al. COVID‐19: poor outcomes in patients with zinc deficiency. Int J Infect Dis. 2020;100:343‐349. 32920234 10.1016/j.ijid.2020.09.014 PMC7482607

[27] Almasaud AS , Chalabi J , Arfaj AA , et al. Association of serum zinc and inflammatory markers with the severity of COVID‐19 infection in adult patients. Nutrients. 2023;15(2):340. 36678211 10.3390/nu15020340 PMC9861200

[28] Pvsn KK , Tomo S , Purohit P , et al. Comparative analysis of serum zinc, copper and magnesium level and their relations in association with severity and mortality in SARS‐CoV‐2 patients. Biol Trace Elem Res. 2023;201(1):23‐30. 35064475 10.1007/s12011-022-03124-7 PMC8782674

[29] Bayraktar N , Bayraktar M , Ozturk A , Ibrahim B . Evaluation of the relationship between aquaporin‐1, hepcidin, zinc, copper, and iron levels and oxidative stress in the serum of critically ill patients with COVID‐19. Biol Trace Elem Res. 2022;200(12):5013‐5021. 36001235 10.1007/s12011-022-03400-6 PMC9399591

[30] Bagher Pour O , Yahyavi Y , Karimi A , et al. Serum trace elements levels and clinical outcomes among Iranian COVID‐19 patients. Int J Infect Dis. 2021;111:164‐168. 34454118 10.1016/j.ijid.2021.08.053 PMC8384760

[31] Ghanei E , Baghani M , Moravvej H , Talebi A , Bahmanjahromi A , Abdollahimajd F . Low serum levels of zinc and 25‐hydroxyvitmain D as potential risk factors for COVID‐19 susceptibility: a pilot case‐control study. Eur J Clin Nutr. 2022;76(9):1297‐1302. 35322170 10.1038/s41430-022-01095-5 PMC8941827

[32] Fromonot J , Gette M , Ben Lassoued A , Guéant JL , Guéant‐Rodriguez RM , Guieu R . Hypozincemia in the early stage of COVID‐19 is associated with an increased risk of severe COVID‐19. Clin Nutr. 2022;41(12):3115‐3119. 34134916 10.1016/j.clnu.2021.04.042 PMC8093004

[33] Allard L , Ouedraogo E , Molleville J , et al. Malnutrition: percentage and association with prognosis in patients hospitalized for coronavirus disease 2019. Nutrients. 2020;12(12):3679. 33260603 10.3390/nu12123679 PMC7761464

[34] Beigmohammadi MT , Bitarafan S , Abdollahi A , et al. The association between serum levels of micronutrients and the severity of disease in patients with COVID‐19. Nutrition. 2021;91‐92:111400. 10.1016/j.nut.2021.111400 PMC8223004 34388583

[35] Dubourg G , Lagier JC , Brouqui P , et al. Low blood zinc concentrations in patients with poor clinical outcome during SARS‐CoV‐2 infection: is there a need to supplement with zinc COVID‐19 patients? J Microbiol Immunol Infect. 2021;54(5):997‐1000. 33632620 10.1016/j.jmii.2021.01.012 PMC7881284

[36] Heller RA , Sun Q , Hackler J , et al. Prediction of survival odds in COVID‐19 by zinc, age and selenoprotein P as composite biomarker. Redox Biol. 2021;38:101764. 33126054 10.1016/j.redox.2020.101764 PMC7574778

[37] Yasui Y , Yasui H , Suzuki K , et al. Analysis of the predictive factors for a critical illness of COVID‐19 during treatment—relationship between serum zinc level and critical illness of COVID‐19. Int J Infect Dis. 2020;100:230‐236. 32911042 10.1016/j.ijid.2020.09.008 PMC7476566

[38] Zeng HL , Yang Q , Yuan P , Wang X , Cheng L . Associations of essential and toxic metals/metalloids in whole blood with both disease severity and mortality in patients with COVID‐19. FASEB J. 2021;35(3):e21392. 33577131 10.1096/fj.202002346RR PMC7995111

[39] Razeghi Jahromi S , Moradi Tabriz H , Togha M , et al. The correlation between serum selenium, zinc, and COVID‐19 severity: an observational study. BMC Infect Dis. 2021;21(1):899. 34479494 10.1186/s12879-021-06617-3 PMC8414458

[40] Shakeri H , Azimian A , Ghasemzadeh‐Moghaddam H , et al. Evaluation of the relationship between serum levels of zinc, vitamin B12, vitamin D, and clinical outcomes in patients with COVID‐19. J Med Virol. 2022;94(1):141‐146. 34406674 10.1002/jmv.27277 PMC8426973

[41] Rodelgo Jimenez L , Anchuelo AC , Soler PM , et al. Zinc levels of patients with a moderate to severe COVID‐19 infection at hospital admission and after 4th days of ward hospitalization and their clinical outcome. J Trace Elem Med Biol. 2023;79:127200. 37229980 10.1016/j.jtemb.2023.127200 PMC10181947

[42] Du Laing G , Petrovic M , Lachat C , et al. Course and survival of COVID‐19 patients with comorbidities in relation to the trace element status at hospital admission. Nutrients. 2021;13(10):3304. 34684306 10.3390/nu13103304 PMC8541297

[43] Gonçalves TJM , Gonçalves SEAB , Guarnieri A , et al. Association between low zinc levels and severity of acute respiratory distress syndrome by new coronavirus SARS‐CoV‐2. Nutr Clin Pract. 2021;36(1):186‐191. 33368619 10.1002/ncp.10612

[44] Tomasa‐Irriguible TM , Bielsa‐Berrocal L , Bordejé‐Laguna L , et al. Low levels of few micronutrients may impact COVID‐19 disease progression: an observational study on the first wave. Metabolites. 2021;11(9):565. 34564381 10.3390/metabo11090565 PMC8467487

[45] Im JH , Je YS , Baek J , Chung MH , Kwon HY , Lee JS . Nutritional status of patients with COVID‐19. Int J Infect Dis. 2020;100:390‐393. 32795605 10.1016/j.ijid.2020.08.018 PMC7418699

[46] Verschelden G , Noeparast M , Noparast M , et al. Plasma zinc status and hyperinflammatory syndrome in hospitalized COVID‐19 patients: an observational study. Int Immunopharmacol. 2021;100:108163. 34583122 10.1016/j.intimp.2021.108163 PMC8450071

[47] Joulaei H , Keshani P , Foroozanfar Z , et al. Serum zinc associated with immunity and inflammatory markers in Covid‐19. Open Med. 2022;17(1):702‐711. 10.1515/med-2022-0469 PMC8990765 35480398

[48] Lahaye C , Parant F , Haesebaert J , et al. Minerals and antioxidant micronutrients levels and clinical outcome in older patients hospitalized for COVID‐19 during the first wave of the pandemic. Nutrients. 2023;15(6):1516. 36986247 10.3390/nu15061516 PMC10056386

[49] Vogel‐González M , Talló‐Parra M , Herrera‐Fernández V , et al. Low zinc levels at admission associates with poor clinical outcomes in SARS‐CoV‐2 infection. Nutrients. 2021;13(2):562. 33572045 10.3390/nu13020562 PMC7914437

[50] Wozniak H , Le Terrier C , Primmaz S , et al. Association of trace element levels with outcomes in critically ill COVID‐19 patients. Nutrients. 2023;15(15):3308. 37571249 10.3390/nu15153308 PMC10421129

[51] Sobczyk MK , Gaunt TR . The effect of circulating zinc, selenium, copper and vitamin K(1) on COVID‐19 outcomes: a mendelian randomization study. Nutrients. 2022;14(2):233. 35057415 10.3390/nu14020233 PMC8780111

[52] Vincent JL . International study of the prevalence and outcomes of infection in intensive care units. JAMA. 2009;302(21):2323‐2329. 19952319 10.1001/jama.2009.1754

[53] Bonanad C , García‐Blas S , Tarazona‐Santabalbina F , et al. The effect of age on mortality in patients with COVID‐19: a meta‐analysis with 611,583 subjects. J Am Med Dir Assoc. 2020;21(7):915‐918. 32674819 10.1016/j.jamda.2020.05.045 PMC7247470

[54] Romero Starke K , Reissig D , Petereit‐Haack G , Schmauder S , Nienhaus A , Seidler A . The isolated effect of age on the risk of COVID‐19 severe outcomes: a systematic review with meta‐analysis. BMJ Glob Health. 2021;6(12):e006434. 10.1136/bmjgh-2021-006434 PMC8678541 34916273

[55] Carlucci PM , Ahuja T , Petrilli C , Rajagopalan H , Jones S , Rahimian J . Zinc sulfate in combination with a zinc ionophore may improve outcomes in hospitalized COVID‐19 patients. J Med Microbiol. 2020;69(10):1228‐1234. 32930657 10.1099/jmm.0.001250 PMC7660893

[56] Partap U , Sharma KK , Marathe Y , et al. Vitamin D and zinc supplementation to improve treatment outcomes among COVID‐19 patients in India: results from a double‐blind randomized placebo‐controlled trial. Curr Dev Nutr. 2023;7(8):101971. 37560461 10.1016/j.cdnut.2023.101971 PMC10407567

[57] Adrean SD , Schmitt K , Ng C , Pirouz A , Ramkumar HL , Grant S . Does prophylactic oral zinc reduce the risk of contracting COVID‐19? Cureus. 2022;14(10):e30881. 36337789 10.7759/cureus.30881 PMC9618326

[58] Gordon AM , Hardigan PC . A case‐control study for the effectiveness of oral zinc in the prevention and mitigation of COVID‐19. Front Med (Lausanne). 2021;8:756707. 34966750 10.3389/fmed.2021.756707 PMC8711630

[59] Thomas S , Patel D , Bittel B , et al. Effect of high‐dose zinc and ascorbic acid supplementation vs usual care on symptom length and reduction among ambulatory patients with SARS‐CoV‐2 infection: the COVID A to Z randomized clinical trial. JAMA Netw Open. 2021;4(2):e210369. 33576820 10.1001/jamanetworkopen.2021.0369 PMC7881357

[60] Age‐Related Eye Disease Study Research Group The effect of five‐year zinc supplementation on serum zinc, serum cholesterol and hematocrit in persons randomly assigned to treatment group in the age‐related eye disease study: AREDS Report No. 7. J Nutr. 2002;132(4):697‐702. 11925463 10.1093/jn/132.4.697 PMC1464086

[61] Lowe NM , Medina MW , Stammers AL , et al. The relationship between zinc intake and serum/plasma zinc concentration in adults: a systematic review and dose‐response meta‐analysis by the EURRECA Network. Br J Nutr. 2012;108(11):1962‐1971. 23244547 10.1017/S0007114512004382

[62] Patel O , Chinni V , El‐Khoury J , et al. A pilot double‐blind safety and feasibility randomized controlled trial of high‐dose intravenous zinc in hospitalized COVID‐19 patients. J Med Virol. 2021;93(5):3261‐3267. 33629384 10.1002/jmv.26895 PMC8014767

[63] Bonaventura P , Benedetti G , Albarède F , Miossec P . Zinc and its role in immunity and inflammation. Autoimmun Rev. 2015;14(4):277‐285. 25462582 10.1016/j.autrev.2014.11.008

[64] Gammoh N , Rink L . Zinc in infection and inflammation. Nutrients. 2017;9(6):624. 28629136 10.3390/nu9060624 PMC5490603

[65] Besecker BY , Exline MC , Hollyfield J , et al. A comparison of zinc metabolism, inflammation, and disease severity in critically ill infected and noninfected adults early after intensive care unit admission. Am J Clin Nutr. 2011;93(6):1356‐1364. 21525204 10.3945/ajcn.110.008417 PMC3095505

[66] Kim YS , Escobar GJ , Halpern SD , Greene JD , Kipnis P , Liu V . The natural history of changes in preferences for life‐sustaining treatments and implications for inpatient mortality in younger and older hospitalized adults. J Am Geriatr Soc. 2016;64(5):981‐989. 27119583 10.1111/jgs.14048 PMC4882256

[67] Stream S , Nolan A , Kwon S , Constable C . Factors associated with combined do‐not‐resuscitate and do‐not‐intubate orders: a retrospective chart review at an urban tertiary care center. Resuscitation. 2018;130:1‐5. 29935341 10.1016/j.resuscitation.2018.06.020 PMC6481924

[68] Wilson ME , Mittal A , Karki B , et al. Do‐not‐intubate orders in patients with acute respiratory failure: a systematic review and meta‐analysis. Intensive Care Med. 2020;46(1):36‐45. 31659387 10.1007/s00134-019-05828-2 PMC7223954

[69] Hennigar SR , Lieberman HR , Fulgoni VL , McClung JP . Serum zinc concentrations in the us population are related to sex, age, and time of blood draw but not dietary or supplemental zinc. J Nutr. 2018;148(8):1341‐1351. 29947812 10.1093/jn/nxy105

[70] King JC . Yet again, serum zinc concentrations are unrelated to zinc intakes. J Nutr. 2018;148(9):1399‐1401. 30184229 10.1093/jn/nxy190

[71] Clougherty JE , Kinnee EJ , Cardet JC , et al. Geography, generalisability, and susceptibility in clinical trials. Lancet Respir Med. 2021;9(4):330‐332. 33539731 10.1016/S2213-2600(21)00046-1 PMC8009610

[72] Yusuf S , Wittes J . Interpreting geographic variations in results of randomized, controlled trials. N Engl J Med. 2016;375(23):2263‐2271. 27959693 10.1056/NEJMra1510065

[73] Pocock S , Calvo G , Marrugat J , et al. International differences in treatment effect: do they really exist and why? Eur Heart J. 2013;34(24):1846‐1852. 23475529 10.1093/eurheartj/eht071

