# Supplementary material for: Serum zinc level independently predicts need for inpatient intubation among patients hospitalized with COVID‐19: A prospective observational study
Source: Nutr Clin Pract. 2025 Nov 11;41(2):608–18. doi: 10.1002/ncp.70070 (PMC12667602; doi:10.1002/ncp.70070)
Supplement: Supplementary file 1 — Table S1: Pilot data primary outcome statistics. Table S2: Estimation of PaO2 from measured SpO2 (EPIC II study estimation table). Table S3: Correlation between serum zinc level, and levels of C‐reactive protein and ferritin. Table S4: Multivariable analysis of factors contributing to discharge to home as the outcome of hospital stay in COVID‐19 infected individuals. [file NCP-41-608-s001.docx]

**Supplemental Materials: Serum zinc level independently predicts need for inpatient intubation among patients hospitalized with COVID-19**

**TABLE OF CONTENTS**

| Supplemental materials and methods | Page 3 |
| --- | --- |
|  |  |
| Table S1 | Page 6 |
|  |  |
| Table S2 | Page 7 |
|  |  |
| Table S3 | Page 8 |
|  |  |
| Table S4 | Page 9 |
|  |  |
|  |  |
|  |  |
|  |  |

**SUPPLEMENTAL MATERIALS AND METHODS**

**Study Design and Settings**

This was a prospective observational study examining whether serum zinc level at hospital admission correlated with disease severity and course in COVID-19 infection. This study was designed in two phases. In the initial pilot phase, a dataset from subjects admitted with COVID-19 infection (intended to number 30) was used to estimate the strength of correlation between serum zinc level and clinical outcomes and make statistical power estimates. In the second phase, these estimates were used to expand the data set to a cohort suitable for statistical analysis. The study was performed at four hospitals including one tertiary academic medical center, two community hospitals, and one COVID-19 specialty hospital.

**Study Population and Timeframe**

This study recruited subjects from July 2020 through June 2022. Inclusion criteria were age 18 years or greater, COVID-19 positive by laboratory testing (both ELISA- and PCR-based testing acceptable), scheduled lab draw within seven days of hospital admission, and admission to a participating hospital. Exclusion criteria were pregnancy and prisoner status. To ensure that statistical power calculations were based on a cohort with a broad range of clinical severity, the initial pilot phase of the study was protocolized such that half of enrollees required respiratory support of at least six liters oxygen by nasal cannula at the time of enrollment and half did not. This resulted in some patients who were otherwise eligible being excluded if the quota for their respiratory status had already been met. The quotas for respiratory support requirements were not continued in the second phase of the study.

**Subject Enrollment**

Due to the infectious hazards posed by COVID-19 and limited availability of personal protective equipment early in the pandemic, we approached and consented eligible patients or their legally authorized representatives (LARs) by telephone only. Study participation, protocol, risks and benefits were described using a pre-written script and all questions were asked and answered prior to enrollment. In light of the concerns above and minimal risk for harm our study posed for subjects, a waiver of written/signed documentation of consent was approved by our IRB, but a brief note outlining the consent discussion and provision of verbal consent was placed in the medical record of each consenting subject. This study was approved by our institution’s Institutional Review Board (STUDY #00009471), which has jurisdiction over all participating hospitals.

**Data Collection**

Serum zinc levels were determined within seven days of admission utilizing existing phlebotomy and clinical laboratory protocols at each hospital. Serum was collected in a metal-free tube, and all participating hospitals sent specimens to same central lab (ARUP Laboratories, Salt Lake City, UT, USA) for serum zinc assessment. To minimize the harm of venipuncture, and reduce exposure risks between subjects and staff, all study lab draws were scheduled to coincide with lab draws that were required for clinical care. All clinical data was extracted from the electronic medical record. The primary outcome for this study was requirement for intubation and mechanical ventilation at any point during hospitalization. Secondary outcomes included respiratory status on hospital day 1 and anytime prior to hospital day 8, presence of shock on hospital day 1 and anytime prior to hospital day 8, presence of acute kidney injury on hospital day 1 or anytime prior to hospital day 8, hospitalization outcome (discharged to home, discharged to facility or deceased), and occurrence of venous thromboembolism. In the event of missing data, the subject was excluded from the associated analysis.

Data was collected to calculate sequential organ failure assessment (SOFA) scores; however, serum bilirubin was not measured for the majority of subjects, so a modified SOFA score including the cardiac, neurologic, hematologic, renal and respiratory subscores, but excluding the hepatic subscore, was used. When partial pressures of arterial oxygen (PaO2) were unavailable they were estimated from peripheral oxygen saturation (SpO_2_) using the EPIC II study estimation table (Table S2).

**Statistical Analysis**

Using preliminary data on serum zinc levels from our pilot group, and assuming that approximately one patient in three would require intubation (also observed during pilot data collection), one-way ANOVA analysis (POWER procedure, SAS 9.4) indicated that a total of 75 subjects would be required to show a statistically significant difference in serum zinc levels between the intubated and un-intubated groups with 80% or greater power. We therefore set our enrollment target at 105 subjects to allow for attrition.

To compare zinc levels between outcomes, we used Wilcoxon rank sum tests. Tests of correlation were performed by calculating Spearman’s rank correlation coefficients and associated p-values. For multivariable analyses, our outcomes were dichotomous so we used logistic regression models. SAS 9.4 was used for all analyses.

**Table S1:** Pilot data primary outcome statistics

| **Required intubation during hospitalization?** | **N** | **Serum zinc level (ug/dL), median [IQR]** |
| --- | --- | --- |
| Yes | 11 | 51.2 [46.1, 59.1] |
| No | 18 | 58.4 [52.2, 80.2] |

**Table S2:** Estimation of PaO2 from measured SpO_2_ (EPIC II study estimation table)

| **Estimating PaO_2_ from SpO_2_** |  |  |
| --- | --- | --- |
| **SpO_2_ (%)** | **Estimated PaO_2_ (mmHg)** |  |
| 80 | 44 |  |
| 81 | 45 |  |
| 82 | 46 |  |
| 83 | 47 |  |
| 84 | 49 |  |
| 85 | 50 |  |
| 86 | 52 |  |
| 87 | 53 |  |
| 88 | 55 |  |
| 89 | 57 |  |
| 90 | 60 |  |
| 91 | 62 |  |
| 92 | 65 |  |
| 93 | 69 |  |
| 94 | 73 |  |
| 95 | 79 |  |
| 96 | 86 |  |
| 97 | 96 |  |
| 98 | 112 |  |
| 99 | 145 |  |
|  |  |  |
| **Estimating FiO_s_** |  |  |
| **Method** | **O_2_ flow (L/min)** | **Estimated FiO_2_ (%)** |
| Nasal Cannula | 1 | 24 |
|  | 2 | 28 |
|  | 3 | 32 |
|  | 4 | 36 |
|  | 5 | 40 |
|  | 6 | 44 |
| Face Mask | 5 | 40 |
|  | 6-7 | 50 |
|  | 7-8 | 60 |

**Table S3:** Correlation between serum zinc level, and levels of C-reactive protein and ferritin.

|  | **Spearman Correlation Coefficient**  **Prob > \|r\| under H0: Rho = 0**  **N** |
| --- | --- |
|  | **Serum zinc level** |
| **C-reactive protein level** | 0.10449  0.3355  87 |
| **Ferritin level** | 0.04315  0.8177  31 |

**Table S4:** Multivariable analysis of factors contributing to discharge to home as the outcome of hospital stay in COVID-19 infected individuals

| **Covariate** | **Odds Ratio (95% CI)** | **P-value** |
| --- | --- | --- |
| Serum zinc level | 0.991 (0.949, 1.034) | p = 0.675 |
| Age | 0.956 (0.917, 0.998) | p = 0.038 |
| Male sex | 0.936 (0.273, 3.211) | p = 0.917 |
| Race non-Caucasian | 1.382 (0.306, 6.250) | p = 0.674 |
| Modified SOFA (mSOFA) score | 0.586 (0.447, 0.767) | p < 0.001 |
| History of hypertension | 0.783 (0.204, 3.0008) | p = 0.721 |
| History of diabetes mellitus (type 1 or 2) | 0.372 (0.095, 1.463) | p = 0.157 |
| History of lung disease | 1.376 (0.341, 5.547) | p = 0.654 |
