# Supplementary material for: Serum zinc level independently predicts need for inpatient intubation among patients hospitalized with COVID‐19: A prospective observational study
Source: Nutr Clin Pract. 2025 Nov 11;41(2):608–18. doi: 10.1002/ncp.70070 (PMC12667602; doi:10.1002/ncp.70070)
Supplement: Supplementary file 2 — McPherson 2025. [file NCP-41-608-s002.pptx]

## Slide 1
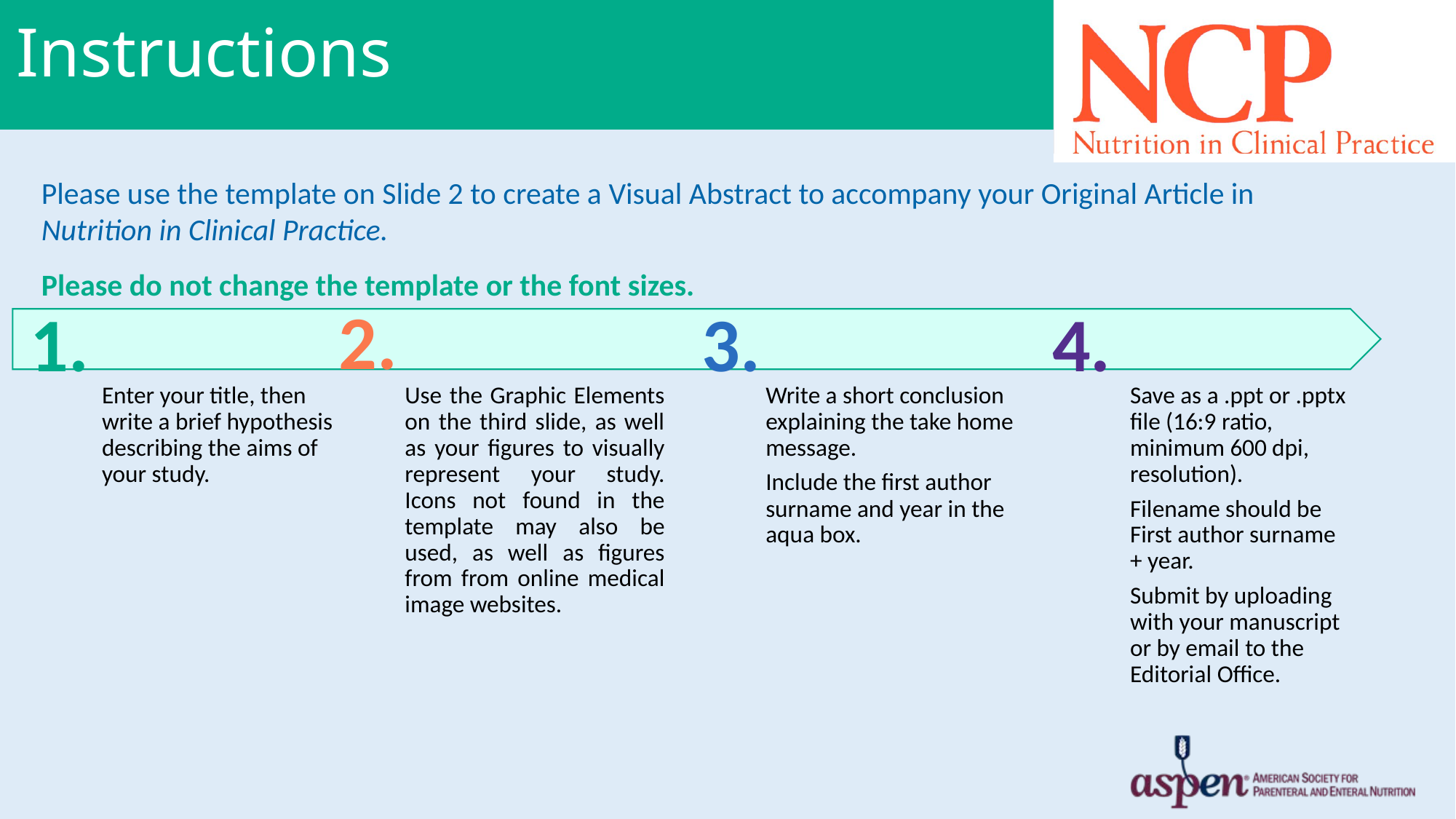

Instructions
Please use the template on Slide 2 to create a Visual Abstract to accompany your Original Article in Nutrition in Clinical Practice.
Please do not change the template or the font sizes.
2.
1.
3.
4.
Save as a .ppt or .pptx file (16:9 ratio, minimum 600 dpi, resolution).
Filename should be First author surname + year.
Submit by uploading with your manuscript or by email to the Editorial Office.
Enter your title, then write a brief hypothesis describing the aims of your study.
Use the Graphic Elements on the third slide, as well as your figures to visually represent your study. Icons not found in the template may also be used, as well as figures from from online medical image websites.
Write a short conclusion explaining the take home message.
Include the first author surname and year in the aqua box.

## Slide 2
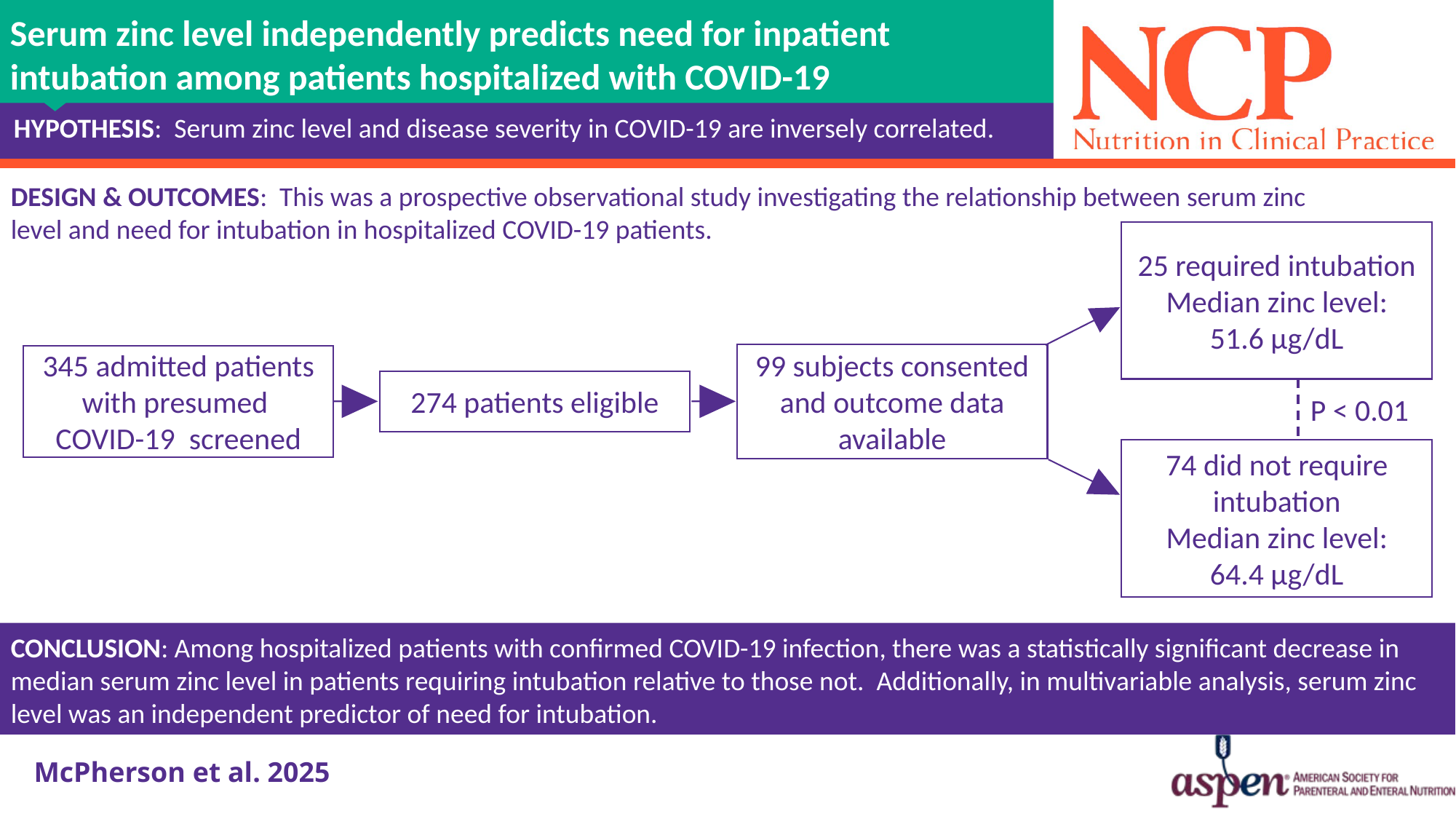

Serum zinc level independently predicts need for inpatient intubation among patients hospitalized with COVID-19
HYPOTHESIS: Serum zinc level and disease severity in COVID-19 are inversely correlated.
DESIGN & OUTCOMES: This was a prospective observational study investigating the relationship between serum zinc level and need for intubation in hospitalized COVID-19 patients.
25 required intubation
Median zinc level:
51.6 µg/dL
99 subjects consented and outcome data available
345 admitted patients with presumed
COVID-19 screened
274 patients eligible
P < 0.01
74 did not require intubation
Median zinc level:
64.4 µg/dL
CONCLUSION: Among hospitalized patients with confirmed COVID-19 infection, there was a statistically significant decrease in median serum zinc level in patients requiring intubation relative to those not. Additionally, in multivariable analysis, serum zinc level was an independent predictor of need for intubation.
McPherson et al. 2025

## Slide 3
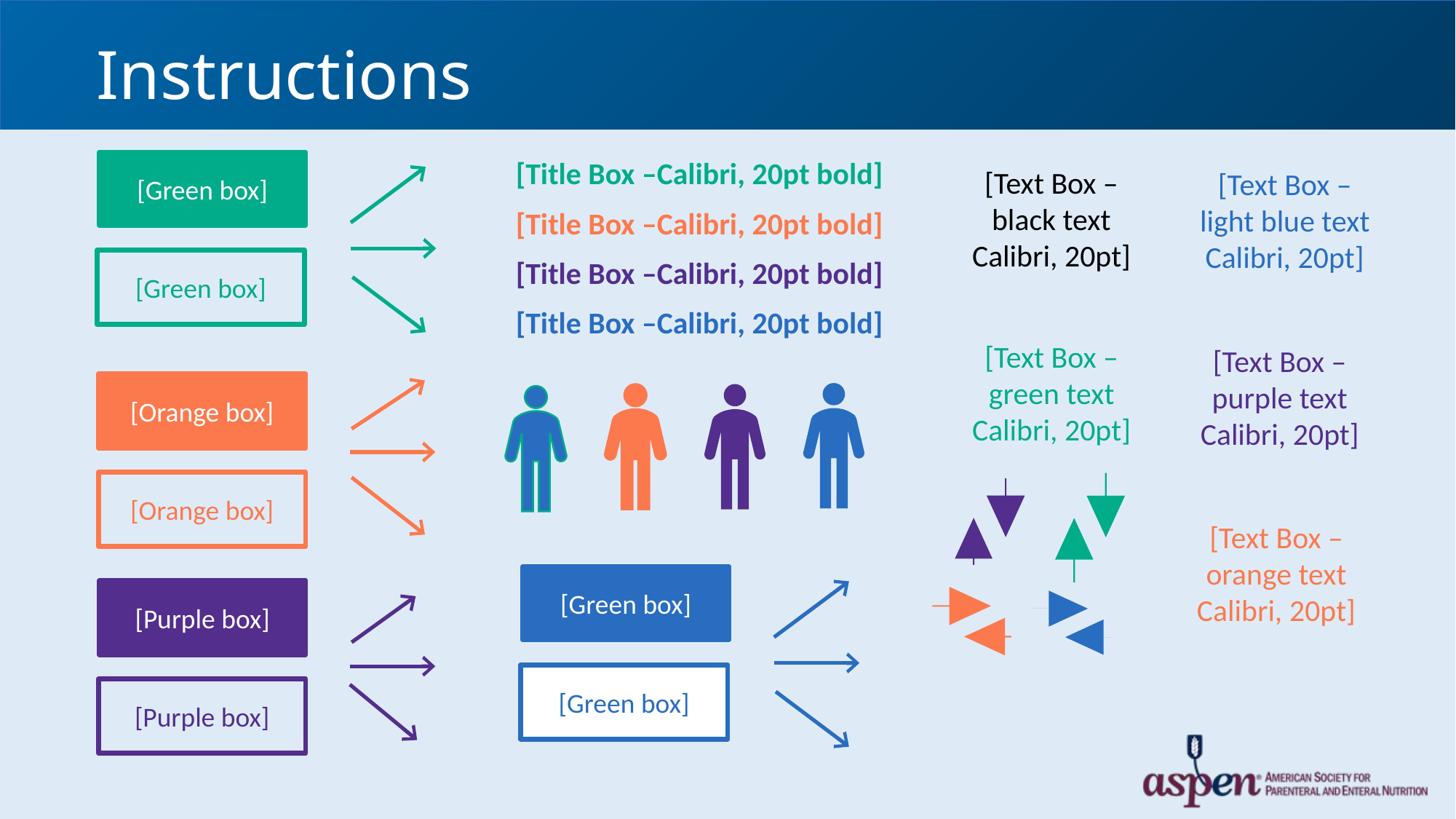

Instructions
[Title Box –Calibri, 20pt bold]
[Green box]
[Text Box – black text Calibri, 20pt]
[Text Box – light blue text Calibri, 20pt]
[Title Box –Calibri, 20pt bold]
[Title Box –Calibri, 20pt bold]
[Green box]
[Title Box –Calibri, 20pt bold]
[Text Box – green text Calibri, 20pt]
[Text Box – purple text Calibri, 20pt]
[Orange box]
[Orange box]
[Text Box –orange text Calibri, 20pt]
[Green box]
[Purple box]
[Green box]
[Purple box]
